# An Evaluation of VYC‐17.5L for Forehead Contouring: A Prospective, Open‐Label, Post‐Marketing Study

**DOI:** 10.1111/jocd.70093

**Published:** 2025-03-03

**Authors:** Sofia Ruiz Del Cueto, Fernando Urdiales Galvez, Alessandro Gritti, Nicola Kefalas, Carola de la Guardia, Graeme Kerson

**Affiliations:** ^1^ Clínica Mira and Cueto Madrid Spain; ^2^ Instituto Médico Miramar Malaga Spain; ^3^ GRITTI Chirurgia Maxillo Facciale Milan Italy; ^4^ Private Practice Torino Italy; ^5^ Allergan Aesthetics, an AbbVie Company Madrid Spain; ^6^ Allergan Aesthetics, an AbbVie Company Marlow UK

**Keywords:** forehead augmentation, forehead contouring, hyaluronic acid, upper third of face, VYC‐17.5L

## Abstract

**Background:**

Forehead contouring can be achieved using injectable fillers. VYC‐17.5L is a filler containing 17.5 mg/mL of hyaluronic acid (HA) with lidocaine (3 mg/mL) for the treatment of skin depressions due to premature aging. Prior studies have demonstrated the safety and efficacy of VYC‐17.5L in nasolabial folds, lips, and radial cheek lines, but little has been published on the effectiveness of VYC17.5L for forehead contouring.

**Aims:**

This prospective, open‐label, post‐marketing study conducted in France evaluated the effectiveness and safety of the HA injectable filler VYC‐17.5L for use in forehead contouring.

**Methods:**

Adults (≥ 18 years) with irregularities of forehead contouring received VYC‐17.5L in the forehead on Day 1 with an optional touch‐up on Day 14. Effectiveness was assessed via investigator‐rated Global Aesthetic Improvement Scale (GAIS) of “improved” or “much improved” from baseline at month 1 (M1). Secondary endpoints included participant‐rated GAIS and FACE‐Q Satisfaction with Facial Appearance. Injection site reactions (ISRs) and adverse device effects (ADEs) were assessed throughout the study.

**Results:**

Eighty subjects completed the study. The primary endpoint was met: 97.5% of participants showed improvement on the investigator GAIS. Participant GAIS scores were also improved. FACE‐Q scores significantly improved from baseline (*p* < 0.0001) and were sustained through month 12. Most ISRs were mild or moderate and resolved in *~*1 week. Nine subjects (11%) reported ADEs, with the majority being mild or moderate; the most common ADE was pain, which resolved in < 15 days.

**Conclusion:**

VYC‐17.5L was well tolerated and effective for improving forehead contouring, as demonstrated by improvements in investigator‐ and participant‐rated measures.

## 1 Introduction

The upper third of the face is one of the first indicators of age, with the forehead being the most prominent. Aging of the forehead involves the loss of fat, increased skin laxity, muscle atrophy, and brow ptosis due to intrinsic and extrinsic aging processes [1]. The effects of aging in the forehead include the development of wrinkles, lack of skin smoothness, and loss of curvature and volume [1, 2]. Forehead contouring treatment may be sought by those individuals seeking a more feminized face due to negative psychosocial impacts or to achieve a smoother facial contour [3].

Cosmetic forehead treatments include invasive surgical techniques such as fat grafting, supraorbital contouring, or hairline adjustment [4, 5]. The risks associated with these procedures are numerous; thus, the most common minimally invasive treatment is the use of dermal fillers due to the lower incidence of complications [6, 7]. There is a paucity of published data on the use of hyaluronic acid (HA) dermal filler to treat the forehead. Forehead contouring, particularly in the supraorbital and lower forehead area, can be modulated using injectable filler. The goals of forehead augmentation are to protect the skin of the forehead anteriorly, thus lifting the brow, and to attenuate the pull of the frontalis muscle on the skin [1]. Bertossi and colleagues evaluated 83 patients over 12 months after injection of HA dermal filler in the forehead, nose, and chin and reported significant improvements in satisfaction with facial appearance with no serious adverse events [8].

Juvederm VOLIFT with lidocaine (VYC‐17.5L; Allergan Aesthetics, an AbbVie company) is a soft tissue filler containing 17.5 mL of HA and 3 mg/mL of lidocaine. The filler is intended for the treatment of skin depression due to conditions such as premature aging. VYC‐17.5L is an ideal choice of filler for forehead contouring because of its rheological and physiochemical properties. More specifically, VYC‐17.5L has a medium‐high elastic modulus, low to medium cohesivity, and a small spread, which allows it to maintain its shape and reduces the chances of displacement from the injected layer in the forehead following injection [9, 10]. The safety and efficacy of VYC‐17.5L have been demonstrated in treating nasolabial folds, lips, and radial cheek lines, but little has been published on its effectiveness on the forehead [11, 12]. Thus, this study aimed to evaluate the efficacy and safety of VYC‐17.5L for the improvement of forehead contouring.

## 2 Materials and Methods

### 2.1 Study Design

**FIGURE 1:**
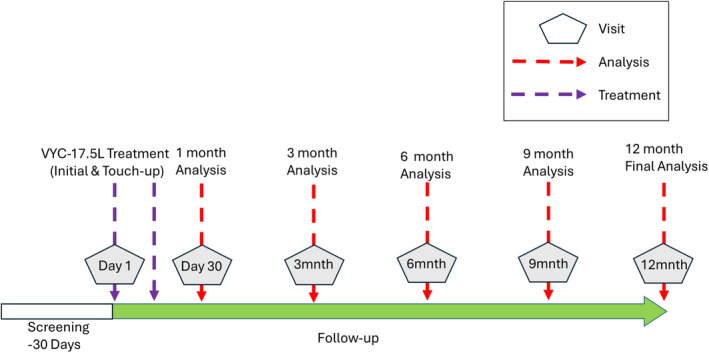
Study Design Schema.

This was a 12‐month, prospective, open‐label, single‐group, post‐marketing study, conducted at 1 site in France from September 2021 through November 2022. This study conformed to the ethical guidelines of the 1975 Declaration of Helsinki. Independent ethics committee approval was obtained from Comite de Protection des Personnes (Lyon, France). All participants provided informed consent prior to any study‐related procedures being performed. This study comprised up to 7 visits: screening (Day −30 to Day 0), baseline visit (Day 1), an optional touch‐up visit (Day 14), and monthly follow‐up thereafter (Months 1, 3, 6, 9, and 12; Figure 1).

### 2.2 Participants

Eligible participants were adults (≥ 18 years of age) that had irregularities of forehead contouring (e.g., protruding orbital rims/frontal bone bossing; above eyebrow depression; central depression; and pan‐forehead depressions) as assessed by the investigator that was likely to see improvement after treatment with VYC‐17.5L. Additional inclusion criteria includedno other medical aesthetic procedures or treatments anywhere in the face not related to the study, negative pregnancy tests before each treatment for women of childbearing potential, and affiliation with a health social system.

Participants were not eligible if they were pregnant or nursing female participants or female participants who planned to get pregnant during the duration of the study; had undergone facial plastic surgery or received permanent facial implants anywhere in the face; had undergone cosmetic resurfacing (i.e., laser, photo‐modulation, intense pulsed light radiofrequency, dermabrasion, chemical peel) anywhere on the face or neck in the last 6 months; used any topical, oral, over‐the‐counter, or prescription anti‐wrinkle products within 90 days of enrollment; any ongoing regimen of anti‐coagulation therapy (e.g., warfarin) or non‐steroidal anti‐inflammatory drugs within 10 days of study treatment; and had an active infection, inflammation (acne, herpes, cancerous or precancerous lesion) or unhealed wound.

### 2.3 Treatment

Treatment was administered following completion of screening, enrollment, and obtaining informed consent from each participant. On Day 1, participants were injected in the forehead at a dosage and injection site determined by the specialist injector (SI) based on their clinical experience. Caution should be used when contouring the forehead with dermal fillers. The injection should occur at least 2 cm above the eyebrow. In addition, the supratrochlear, supraorbital, and superficial temporal vessels should be avoided. Deep injections should be utilized to avoid the subcutaneous blood vessels and nerves. The majority of investigators used a fanning technique or a combination of fanning and a small bolus when administering the treatment. The maximum volume was 2 mL for both the initial and touch‐up visits combined, with a 1 mL maximum allowable volume per treatment session. On Day 14, an optional touch‐up treatment was provided if the investigator and participant determined it was necessary based on the aesthetic results of the first injection. Follow‐up visits occurred following initial treatment at Months 1, 3, 6, 9, and 12, with Month 1 (M1) being the primary evaluation timepoint.

### 2.4 Efficacy Endpoints

The investigator at the site assessed the forehead for contour irregularities in consultation with the participant. The primary efficacy endpoint was the proportion of participants with an investigator‐assessed Global Aesthetic Improvement Scale (GAIS) rating of “improved” or “much improved” via live evaluation at M1 compared to two‐dimensional (2D) baseline photographs. Secondary efficacy endpoints included the following: change in overall FACE‐Q Satisfaction with Facial Appearance scores from baseline to M1, 3, 6, 9, and 12; proportion of participant‐assessed GAIS ratings of “improved” or “much improved” via live evaluation compared to baseline two‐dimensional (2D) photographs at M1, 3, 6, 9, and 12; and volume and contour change in the forehead using three‐dimensional (3D) imaging at M1, 3, 6, 9, and 12 compared to baseline. Image analysis of 3D images captured using the Vectra M3, a photography system that uses high resolution to capture a detailed 3D image of the participant's face, from each visit was used to assess volumetric and contour changes from baseline. 2D photographs for the frontal, 45° left, and 45° right angles were created from the 3D image used for the GAIS evaluation.

### 2.5 Safety Endpoints

Injection site reactions (ISRs) were captured for the first 30 days after treatment utilizing participants' diaries that assessed the tolerance of VYC‐17.5L on a 4‐point numerical scale (scale of 0–3, 0 = none, 1 = mild, 2 = moderate, and 3 = severe). ISRs were not reported as adverse events (AEs) unless they were still present 30 days after injection, required medical intervention, or were considered by the investigator to be abnormal.

### 2.6 Statistical Analyses

Based on a previous study whereby 87% of participants showed improvement on the GAIS at M1 and assuming a response rate of 88% and a 10% attrition rate, 80 participants would be adequate to produce a 95% confidence interval (CI) with a width of 0.15 [13]. The modified intention‐to‐treat (mITT) population consisted of all randomized participants with at least 1 baseline and 1 post‐treatment forehead assessment. Efficacy analyses were performed on the mITT population. The change from baseline in forehead volume was analyzed using paired *t*‐tests. The safety population consisted of all treated participants. All safety analyses were performed on the safety population. All statistical tests were 2‐sided hypothesis tests performed at the 5% significance level using SAS software version 9.4.7.

## 3 Results

### 3.1 Participants

Of the130 screened participants, 80 were enrolled, and 75 (93.7%) completed the study. Reasons for discontinuing included the following: withdrawal by the subject (*n* = 3), lost to follow‐up (*n* = 1), and incidence of an adverse event (*n* = 1). The mean age of participants was 55.4 years. The majority of participants were female (91.3%) and Fitzpatrick skin phototype II (41.3%; Table 1). The average injection volume was 0.92 mL for the initial treatment and 0.56 mL for those participants (*n* = 26; 32.5%) who received a touch‐up injection.

**TABLE 1:** Participant demographics (safety population).

Characteristic	Forehead contouring (*n* = 80)
Age, years	
Mean (SD)	55.4 (10.7)
Median	56.5
Range	28–70
Sex, *n* (%)	
Female	73 (91.3)
Male	7 (8.8)
Fitzpatrick skin phototype, *n* (%)	
I	1 (1.3)
II	33 (41.3)
III	28 (35.0)
IV	18 (22.5)

*Note:* Participants were treated at a single site in France.

Abbreviation: SD, standard deviation.

### 3.2 Efficacy: Investigator and Participant GAIS and FACE‐Q Scores

**FIGURE 2:**
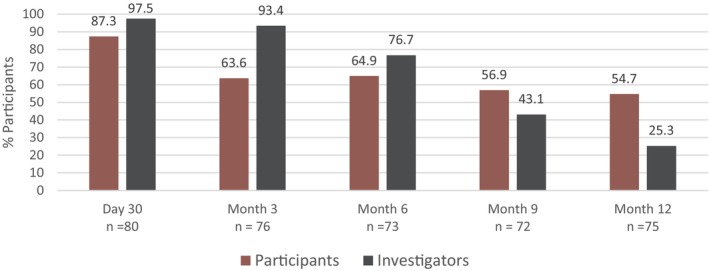
Proportion of Responders Achieving Investigator and Participant‐Assessed GAIS rating of Improved or Much Improved Over 12 Months After VYC‐17.5L Treatment. GAIS, Global Aesthetic Improvement Scale.

**FIGURE 3:**
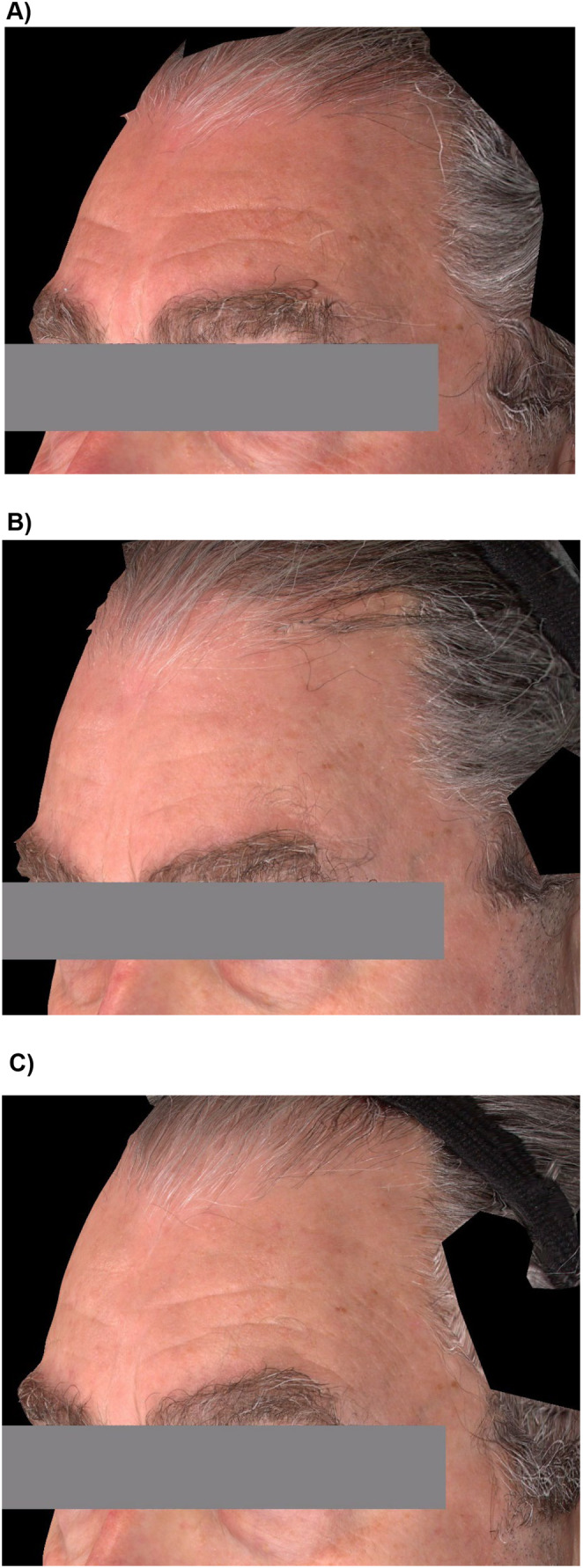
Representative Photographs of Participants after Treatment with VYC‐17.5L at Baseline (A), Month 3 (B) and Month 12 (C).The participant was a 67‐year‐old male who received 1 mL of VYC‐17.5L supraperiosteally to the forehead. On Day 14, a 0.85mL touch‐up was administered subdermally.

The primary endpoint was met with 97.5% of participants being rated as “improved” or “much improved” on the investigator‐assessed GAIS. The high rating continued through M3 before declining from M6 through M12 to 25.3% (Figure 2). The participant‐assessed GAIS ratings were comparable to the investigator GAIS but were lower at M1, with 87.3% rating their foreheads as “improved” or “much improved” after VYC‐17.5L treatment (Figure 2). The ratings remained higher than on the investigator GAIS, with just over half of participants (54.7%) indicating the improvement was still present at M12. Representative photographs of participants throughout the course of the study are presented in Figure 3.

**FIGURE 4:**
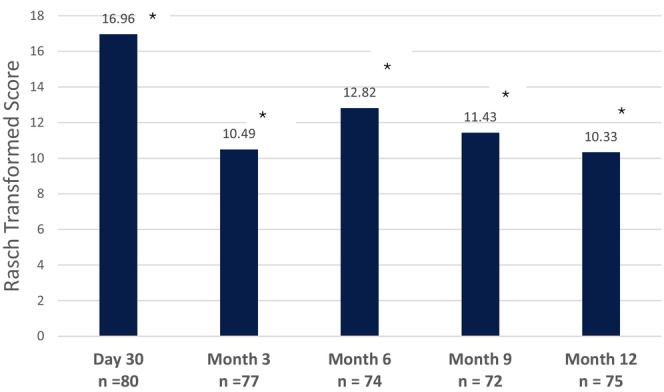
Change from Baseline in FACE‐Q Satisfaction with Facial Appearance Scores Over the Course of the Study. *p < 0.0001 for the Wilcoxon test.

**FIGURE 5:**
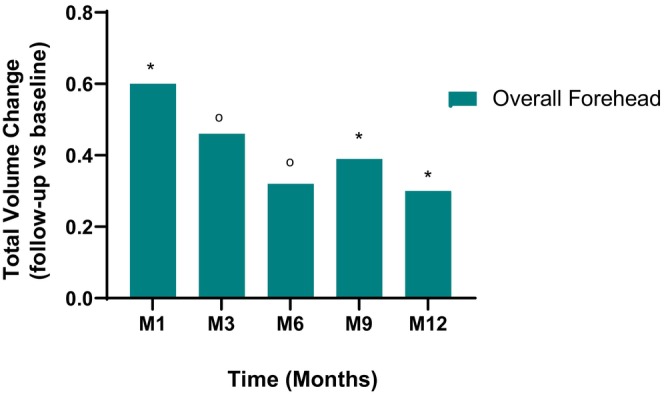
Total Volume Change from Baseline in Forehead of Participants Treated with VYC‐17.5L Over 12 Months. M1, 3, 6, 9, 12 = Month 1, 3, 6, 9, and 12. *p < 0.0001 for the Wilcoxon test; ^O^ *p* < 0.0001 for the paired Student's *t*‐test.

The change in the mean Rasch transformed FACE‐Q satisfaction with facial appearance total score from baseline was 16.96 at M1 (*p* < 0.0001; Figure 4). The mean change in score declined over time from 10.49 at M3 to 10.33 at M12 (*p* < 0.0001 for all timepoints). Lastly, a significant increase in the total volume change was observed at M1 via 3D photography (*p* < 0.0001; Figure 5). The improvement in volume was maximal at M1 and progressively decreased through M12 but remained significant (*p* < 0.0001 for overall forehead at all timepoints).

### 3.3 Safety

The most prevalent ISRs were pain/tenderness (76%), lumps/bumps (46%), induration (42%), and swelling/edema (41%). The most frequently reported ISRs were pain/tenderness. Most ISRs were mild or moderate and resolved spontaneously within 1 week; the mean duration for the majority of ISRs was 1–3 days. One participant experienced a serious adverse event (SAE) unrelated to the treatment, a discovery of pancreatic cancer, and withdrew from the study. Nine adverse device effects (ADEs) were reported in 9 participants (Table 2). The most frequently reported ADEs were as follows: pain (66.7%), mass (22.2%), and urticaria (11.1%). Most ADEs lasted < 2 weeks and resolved spontaneously. No serious ADEs were reported.

**TABLE 2:** Summary of ADEs Occurring Among Participants Treated with VYC‐17.5L.

ADE category	Forehead contouring (*n* = 9)
Participants with at least 1 ADE, *n* (%)	9 (100.0)
General disorders and administration site conditions, *n* (%)	
Injection site hematoma	0 (0.0)
Injection site irritation	0 (0.0)
Injection site mass	2 (22.2)
Injection site nodule	0 (0.0)
Injection site pain	6 (66.7)
Injection site urticaria	1 (11.1)

Abbreviation: ADE, adverse device effects.

## 4 Discussion

This 12‐month prospective, single‐center study demonstrated significant improvement in forehead contouring after treatment with up to 2 mL of VYC‐17.5L. Investigator and participant assessments via live evaluation remained high throughout the study when compared to 2D baseline photographs. In addition, patient satisfaction ratings were high, and VYC‐17.5L treatment‐related ISRs and ADEs were mostly localized and spontaneously resolved.

Previous studies have evaluated the effect of dermal fillers on forehead contouring. In a 24‐month study assessing the efficacy and safety of a polycaprolactone (PCL)‐based dermal filler containing lidocaine, researchers found that GAIS scores were highest from 1 to 3 months post‐treatment and were sustained, albeit at lower scores, through the end of the study [14]. This aligns with the current study, as a similar pattern was observed in investigator and participant GAIS scores over 12 months.

One limitation of this study is the lack of phototype diversity among participants. Over 76% of participants were classified as having a skin phototype of II or III, limiting the generalizability of the results. The study was only conducted at 1 site in France; repeating the study in other countries with varied ethnicities and skin phototypes, such as East Asia where forehead contouring is a more common aesthetic concern, would provide more evidence of the efficacy and safety of VYC‐17.5L in other populations. Finally, a validated measure of forehead contouring is not currently available, so a validated endpoint was not used in the current study. However, data collected in this study may be used to develop a validated forehead contouring measure for use in future studies.

## 5 Conclusion

Forehead contouring with VYC‐17.5L is effective and well tolerated over 12 months.

## Author Contributions

All authors contributed to the conception and design of the paper and the interpretations provided; were involved in drafting the manuscript and revising it critically for important intellectual content; gave final approval of the version to be published; and agreed to be accountable for all aspects of the work.

## Ethics Statement

The authors confirm that independent ethics committee approval was obtained from Comite de Protection des Personnes (Lyon, France). Participants provided informed consent prior to the initiation of study procedures during the screening visit.

## Conflicts of Interest

Fernando Urdiales‐Galvez investigator for Allergan Aesthetics, an AbbVie company. Alessandro Gritti advisory board member, speaker, and trainer for Allergan Aesthetics, an AbbVie company. Nicola Kefalas advisory board member, speaker, and trainer for Allergan Aesthetics, an AbbVie company. Carola De La Guardia employee of AbbVie and may own AbbVie stock/options. Graeme Kerson employee of AbbVie and may own AbbVie stock/options. Sofia Ruiz Del Cueto the author declares no conflicts of interest.

## Data Availability

AbbVie is committed to responsible data sharing regarding the clinical trials we sponsor. This includes access to anonymized, individual, and trial‐level data (analysis data sets), as well as other information (e.g., protocols, clinical study reports, or analysis plans), as long as the trials are not part of an ongoing or planned regulatory submission. This includes requests for clinical trial data for unlicensed products and indications. These clinical trial data can be requested by any qualified researchers who engage in rigorous, independent, scientific research, and will be provided following the review and approval of a research proposal, Statistical Analysis Plan (SAP), and execution of a Data Sharing Agreement (DSA). Data requests can be submitted at any time after approval in the US and Europe and after acceptance of this manuscript for publication. The data will be accessible for 12 months, with possible extensions considered. For more information on the process or to submit a request, visit the following link: https://www.abbvieclinicaltrials.com/hcp/data‐sharing/html.
